# *In vivo* function and comparative genomic analyses of the *Drosophila* gut microbiota identify candidate symbiosis factors

**DOI:** 10.3389/fmicb.2014.00576

**Published:** 2014-11-04

**Authors:** Peter D. Newell, John M. Chaston, Yiping Wang, Nathan J. Winans, David R. Sannino, Adam C. N. Wong, Adam J. Dobson, Jeanne Kagle, Angela E. Douglas

**Affiliations:** ^1^Department of Entomology, Cornell UniversityIthaca, NY, USA; ^2^Department of Nutritional Science, Cornell UniversityIthaca, NY, USA; ^3^Department of Microbiology, Cornell UniversityIthaca, NY, USA; ^4^Department of Biology, Mansfield UniversityMansfield, PA, USA; ^5^Department of Molecular Biology and Genetics, Cornell UniversityIthaca, NY, USA

**Keywords:** microbiome, symbiosis, genome, *Acetobacter*, *Lactobacillus*

## Abstract

Symbiosis is often characterized by co-evolutionary changes in the genomes of the partners involved. An understanding of these changes can provide insight into the nature of the relationship, including the mechanisms that initiate and maintain an association between organisms. In this study we examined the genome sequences of bacteria isolated from the *Drosophila melanogaster* gut with the objective of identifying genes that are important for function in the host. We compared microbiota isolates with con-specific or closely related bacterial species isolated from non-fly environments. First the phenotype of germ-free *Drosophila* (axenic flies) was compared to that of flies colonized with specific bacteria (gnotobiotic flies) as a measure of symbiotic function. Non-fly isolates were functionally distinct from bacteria isolated from flies, conferring slower development and an altered nutrient profile in the host, traits known to be microbiota-dependent. Comparative genomic methods were next employed to identify putative symbiosis factors: genes found in bacteria that restore microbiota-dependent traits to gnotobiotic flies, but absent from those that do not. Factors identified include riboflavin synthesis and stress resistance. We also used a phylogenomic approach to identify protein coding genes for which fly-isolate sequences were more similar to each other than to other sequences, reasoning that these genes may have a shared function unique to the fly environment. This method identified genes in *Acetobacter* species that cluster in two distinct genomic loci: one predicted to be involved in oxidative stress detoxification and another encoding an efflux pump. In summary, we leveraged genomic and *in vivo* functional comparisons to identify candidate traits that distinguish symbiotic bacteria. These candidates can serve as the basis for further work investigating the genetic requirements of bacteria for function and persistence in the *Drosophila* gut.

## Introduction

All animals are closely associated with microorganisms that are generally not harmful to their animal host. These symbioses can be characterized by co-adaptations that lead to mutual interdependence. This is particularly evident in the partnerships between insects and obligate mutualistic bacteria, where significant genome reduction can occur in vertically inherited bacterial symbionts (McCutcheon and Moran, 2012). In associations where there is the opportunity for horizontal inheritance, such as gut microbiota in animals, the genomic signature of co-evolution may be less overt but still present (Ochman et al., 2010; Frese et al., 2011).

The gut microbiota plays a central role in animal physiology, impacting nutrient acquisition, energy homeostasis, behavior and infection resistance (Smith et al., 2007; Stecher and Hardt, 2008; Cryan and Dinan, 2012; Karasov and Douglas, 2013). Many studies have highlighted the dynamic nature of gut microbial communities, and the significance of taxonomic compositional changes for host health (Turnbaugh et al., 2009; Loh and Blaut, 2012; Lozupone et al., 2012; Karlsson et al., 2013). Thus, an important step toward understanding how a beneficial microbiota is maintained is identifying traits of gut bacteria that contribute to establishment and persistence in the host.

There were two major goals of this study: to describe the phenotypic and genomic characteristics of bacteria isolated from the *Drosophila melanogaster* gut, and to utilize comparative analyses to identify candidate bacterial genes and functional traits that may play a role in this symbiosis. *Drosophila* is a powerful model for studying host-gut microbiota interactions (Broderick and Lemaitre, 2012; Buchon et al., 2013; Erkosar et al., 2013; Lee and Lee, 2014). In particular, taxonomic profiling of the *Drosophila* gut microbiota has revealed it to be a low diversity community, dominated by as few as five OTUs (Chandler et al., 2011; Wong et al., 2011, 2013; Broderick and Lemaitre, 2012; Staubach et al., 2013). Several recent studies have shown that individual gut microbiota isolates can promote larval growth or development rate (Shin et al., 2011; Storelli et al., 2011; Ridley et al., 2012). In a subsequent study, we cultivated five bacterial species corresponding to the dominant OTUs in our laboratory flies (*Acetobacter pomorum, Acetobacter tropicalis, Lactobacillus brevis, Lactobacillus fructivorans*, and *Lactobacillus plantarum*), and introduced them to microbiologically sterile (axenic) animals individually and in combination (Newell and Douglas, 2014). This work demonstrated that the larval development rate and triglyceride content of flies colonized with specific bacteria (gnotobiotic flies) varied depending on the taxon present: mono-colonization with *Acetobacter* species significantly reduced host development time and triglycerides compared to axenic animals while *Lactobacilli* had a limited effect (or no effect) on these traits, depending on the species. Furthermore, we demonstrated that a defined microbiota consisting of all five species supported flies with a development time and nutrient profile comparable to conventionally reared flies (Newell and Douglas, 2014). In the present study, we determined a draft genome sequence for each of the five species to gain insight into the genetic makeup of the *Drosophila* gut microbiota.

Unlike some animal-associated bacterial clades (Brune, 2014; Douglas, 2014), the *Acetobacter* and *Lactobacillus* species present in the *Drosophila* gut have also been isolated from other environments, especially nutrient-rich substrates, e.g., fermented foods. Genome sequences are publicly available for a number of these isolates, including conspecifics or species closely related to each of the five fly isolates we have characterized. The availability of genome-sequenced relatives of our strains presented a unique opportunity to compare the functional traits of bacteria from the *Drosophila* symbiosis and free-living environments. We investigated function from two principal perspectives: by comparing the phenotype of *Drosophila* that are microbiologically sterile (axenic flies) and that are colonized with specific bacteria (gnotobiotic flies); and by sequencing the genomes of the five bacteria isolated from *Drosophila*. Additionally, the metabolic properties were characterized for fly isolates using BioLog plates. These analyses enabled us to test for candidate attributes and genes that may be unique to fly-associated bacteria.

## Materials and methods

### Cultivation of bacteria and flies

*Drosophila melanogaster* Canton S (*Wolbachia*-free) were reared at 25°C, 12 h:12 h light-dark cycle, on yeast-glucose diet: 100 g l^−1^ Brewer's yeast (inactive; MP Biomedicals), 100 g l^−1^ glucose (Sigma), 12 g l^−1^ agar (Apex) and preservatives [0.04% phosphoric acid, 0.42% propionic acid (Sigma)]. *Drosophila* gut microbiota members were isolated on modified MRS agar from aseptically-dissected fly guts. All bacteria used in the study are listed in Table 1, and were maintained at 30°C. Modified MRS contains (all from Sigma unless noted): 1.25% vegetable peptone (Becton Dickinson), 0.75% yeast extract, 2% glucose, 0.5% sodium acetate, 0.2% dipotassium hydrogen phosphate, 0.2% triammonium citrate, 0.02% magnesium sulfate heptahydrate, 0.005% manganese sulfate tetrahydrate, 1.2% agar (Apex). Potato medium contains: 0.5% glucose, 1% yeast extract, 1% peptone, 0.8% potato extract (Fluka 07915), 1.2% agar (Apex). Bacteria we isolated from *Drosophila* guts are identified as ^F^ for “fly isolate” and those isolated from other sources as ^NF^ for “non-fly,” e.g., *Acetobacter tropicalis*^F^ vs. *A. tropicalis*^NF^.

**Table 1:** **Bacterial strains used in this study**.

**Strain**	**Source ^(designation)1^**	**References**
*Lactobacillus plantarum* DmCS_001	*D. melanogaster* gut (^F^)	This study
*Lactobacillus fructivorans* DmCS_002	*D. melanogaster* gut (^F^)	This study
*Lactobacillus brevis* DmCS_003	*D. melanogaster* gut (^F^)	This study
*Acetobacer pomorum* DmCS_004	*D. melanogaster* gut (^F^)	This study
*Acetobacter malorum* DmCS_005	*D. melanogaster* gut (^F^)	This study
*Acetobacter tropicalis* DmCS_006	*D. melanogaster* gut (^F^)	This study
*Acetobacter pasteurianus* NBRC 101655	Pineapple (^NF^) (Thailand)	Chinnawirotpisan et al., 2003
*Acetobacter tropicalis* NBRC 101654	Coconut (^NF^) (Thailand)	Matsutani et al., 2011
*Lactobacillus brevis* ATCC 27305	Wine (^NF^) (Germany)	Nonomura and Ohara, 1967
*Lactobacillus fructivorans* KCTC 3543	Air in dairy barn (^NF^) (Korea)	Nam et al., 2012
*Lactobacillus plantarum* WCFS1	Human saliva (^NF^) (United Kingdom)	Kleerebezem et al., 2003

1 *All fly-derived strains were obtained from a laboratory culture of D. melanogaster (USA)*.

### Bacterial phenotypying by biolog plates

The phenotypic assays comprised utilization of 71 different carbon sources and resistance to 23 chemical stressors using BioLog Gen III MicroPlate (BioLog, Hayward, CA), with tetrazolium redox dyes to quantify metabolic activity. The bacteria were grown in Potato Medium (*Acetobacter*) or Modified MRS (*Lactobacillus*) overnight. Cells were collected by centrifugation, washed once in sterile minimal medium base (18 mM ammonium chloride, 5 mM sodium citrate, 23 mM dibasic sodium phosphate, 1 mM potassium chloride, 2 mM magnesium sulfate), and resuspendend at an OD_600_ of 0.01 in Innoculation Fluid A (BioLog). Then, 100 μl bacterial suspension was transferred to each well of a GenIII microplate (BioLog). After 36 h of incubation at 30°C, color changes in the plate were detected as optical density at 550 nm using a BioRad XMark spectrophotometer, wells A1 and A10 serving as negative and positive controls, respectively (as per manufacturer's instructions). Data were obtained for *A. pomorum^F^*, *A. tropicalis^F^*, *L. brevis*^F^ and *L. plantarum*^F^, but poor growth of *L. fructivorans*^F^ on the Biolog inoculation medium and other defined media tested prevented the implementation of Biolog for this species.

### Preparation of axenic and gnotobiotic flies

Freshly laid eggs (≤18 h old) were collected from grape juice agar plates, and surface sterilized by 3 washes with 0.6% hypochlorite (equivalent to 1:10 dilution of Chlorox bleach) followed by 3 washes with sterile water, and aseptically transferred to sterile food. Inocula for gnotobiotic flies were prepared as follows and added to the food surface after aseptic egg transfer: An overnight culture of each bacterial species used was pelleted and cells were resuspended in fresh growth medium at a final cell density of 10^8^ cells per ml as described (Newell and Douglas, 2014). Fifty μl of cell suspension were added to each gnotobiotic vial to give 5 × 10^6^ cells per vial. For microbiota of >1 species, each component was added in equal parts to make up the total inoculum (e.g., the 5-species microbiota inoculum per vial contained 1 × 10^6^ cells of each species).

### Insect development

Larval development time is a *Drosophila* trait influenced by the gut microbiota (Bakula, 1969; Shin et al., 2011; Storelli et al., 2011; Ridley et al., 2012). To compare the impact of microbiota treatments on development, observations were made three times daily at 0, 6, and 11.5 h after the beginning of the circadian light cycle from initiation of experiments with eggs until puparium formation. For each experimental design, data from 5 independent experiments were collected. Data were analyzed in R Software for Statistical Computing, version 2.15.3 using the Survival, coxme, and multcomp packages following the procedure of Newell and Douglas (2014).

### Nutritional indices

Nutritional contents of adult flies are impacted by the gut microbiota (Shin et al., 2011; Ridley et al., 2012; Newell and Douglas, 2014). To quantify changes in host nutrient content due to microbiota treatments, mated females were collected under light CO_2_ anesthesia 5–6 days post-eclosion, weighed in groups of 3–5 to the nearest μg, using a Mettler Toledo (MX5) microbalance, then homogenized in 125 μl TET buffer (10 mM Tris pH 8, 1 mM EDTA, 0.1% Triton X-100) in 1.5 ml tubes with ~100 μl of lysis matrix D (MP Biomedicals), shaking for 30 s in a FastPrep®−24 instrument on default settings (MP Biomedicals). Next, tubes were centrifuged 1 min at 20 k × g to pellet debris. Twenty μl of the resulting supernatant were flash frozen for subsequent protein determination, while 40 μl were heated at 72°C for 20 min to inactivate endogenous enzymes and subsequently frozen. Replicates for each treatment in each experiment consisted of 3 groups of flies from 3 different vials. A second group of 3–5 flies from each of the 3 vials was collected for CFU determination. Protein content was analyzed using the Bio-Rad DC kit according to manufacturer's instructions. Triglyceride (TAG) was measured using the Free Glycerol Detection Kit in combination with Triglyceride Reagent, following manufacturer's instructions (Sigma). Glucose content was measured by the Glucose Oxidase (GO) method as described previously (Newell and Douglas, 2014).

### Feeding assay

Feeding was quantified by dyed food ingestion, with a procedure modified from Wong et al. (2009). Mated 6–7-day-old adult females were collected 6–10 h after dawn and transferred in groups of 8–10 to 4 empty vials per treatment. After 2 h without food, 3 of the 4 vials were transferred to food containing 0.5% xylene cyanol and 0.1% bromophenol blue (Sigma X4126, and B0126) while the fourth was transferred to food without dye. The vials were frozen at −20°C after a 30 min feeding period. To quantify the food ingested, flies from each vial were washed thoroughly in water, then laid on a paper towel to remove excess moisture. Flies with blue dye visible in their guts were transferred to a microfuge tube, and homogenized as described above for nutritional samples. Following homogenization, an additional 300 μl TET buffer was mixed in and samples centrifuged 3 min at 20 k × g. The absorbance of 200 μl samples of supernatant was quantified at 614 nm in 96-well plate format. The absorbance of flies fed without dye was subtracted as a blank. The remaining absorbance value was related to μg of dyed food by a standard curve generated from dyed food homogenized and measured in the same manner.

### CFU determination

The density of bacteria in whole flies was determined to assess the ability of each bacterial species to associate with the host. Samples of 3–5 female flies were homogenized as described above, except modified MRS medium was employed instead of TET buffer. The resulting homogenates were diluted to 1 ml, and assayed for bacterial abundance by spiral plating (on a WASP-2 instrument, Microbiology International) on modified MRS. CFU counts were made with the Protocol 3 colony counter (Microbiology International).

### Statistical analyses

All statistics were performed in R, version 2.15.3. When ANOVA indicated significant differences, a linear mixed effects model was implemented using the multcomp and lme4 packages with experiment as a random effect. This approach allowed us to account for any “block” variation among experiments. Pairwise comparisons were made via Tukey's test (ghlt function in multcomp, correcting *P*-values for multiple comparisons by the single-step method). Mann-Whitney (MW) pairwise tests were made with the wilcox.test function, and *P*-values were adjusted for multiple comparisons by the Bonferroni correction.

### DNA isolation and sequencing

Bacteria were grown statically (*Lactobacillus*) or shaking (*Acetobacter*) to late-log phase and genomic DNA isolated with the Qiagen DNeasy Blood and Tissue Kit as per the manufacturer's recommendations. Lysozyme digestion was applied to the *Lactobacillus isolates* following the Gram-positive pre-treatment procedure. The Cornell Life Sciences Core Facility performed Illumina library preparation and sequencing using a 100 bp paired-end approach and an Illumina HiSeq 2000 instrument, obtaining 16,320,000–25,000,000 read pairs per genome that passed quality filtering (840–2400X coverage).

We assembled each genome using Velvet 1.2.03 (Zerbino and Birney, 2008). Sequences were randomly divided into subsets (sequence sets) that were estimated to yield a kmer length of 79, (http://dna.med.monash.edu.au/~torsten/velvet_advisor/). Genome sizes were estimated using reference genomes in NCBI, and approximated 100–200X genome coverage. Each sequence set was assembled into contigs using a range of kmer lengths from 51 to 97 (increments of 2). For each sequence set assembly an optimal kmer length was manually selected that: minimized contig #; maximized the N50 score and maximum contig length; and converged upon a common genome coverage across kmer lengths (usually 77, 79, or 81). We also manually trimmed high abundance, low-coverage reads and estimated actual kmer coverage from the assembly. The output contig file from each curated sequence set was used as input in a second Velvet run with all other sequence sets for that genome to create a final assembly (manually curated as above). Annotation and subsequent analyses were performed using the Rapid Annotation using Subsystem Technology (RAST) server (Aziz et al., 2008) to create an annotated genome sequences. The Whole Genome Shotgun projects have been deposited at GenBank under the following accession numbers: *A. pomorum* JOKL00000000; *A. tropicalis*, JOKM00000000; *A. malorum*, JOJU00000000; *L. brevis*, JOKA00000000; *L. frutivorans*, JOJZ00000000; *L. plantarum*, JOJT00000000.

### Comparative analyses

Amino acid sequences from predicted open reading frames in each genome were clustered into clusters of orthologous groups (COGs) *de novo* relative to all available draft or complete genomes of *Acetobacteraceae* (29) or *Lactobacillus* (78) in NCBI (Dec 2013), along with newly generated genome sequences described here. For the *Acetobacteraceae* a draft sequence for *Acetobacter malorum* was also included in this analysis. COGs were called using default instructions for OrthoMCL with an inflation factor of 1.5 (Li et al., 2003). Briefly, amino acid sequence files were extracted from NCBI, or (if no ORFS were available) ORFs were called from nucleotide contig files in RAST. Amino acid sequences were formatted for OrthoMCL and searched against all amino-acid sequences in the taxon pool using a custom sequence database and blastall (blast-2.2.26; database updated Mar 2013). Custom perl scripts were used to redistribute protein sequences from each COG to the respective bacterial taxa to create taxon-specific gene lists and to assist in creating Venn diagrams. A representative gene for each COG was selected using HMMer (Finn et al., 2011). Briefly, an HMM profile for each COG was built from an alignment of all COG sequences created using Muscle (Edgar, 2004). The best match for the HMM profile against all protein sequences from the analyzed taxa (e.g., either of all tested *Acetobacter* or *Lactobacillus* species) was obtained using HMMsearch and selected as a representative sequence for the cluster. The annotation of the selected protein was retained as the annotation for the cluster. Whole genome alignments were performed using MUMmer2, as implemented in the program Jspecies (Kurtz et al., 2004; Richter and Rossello-Mora, 2009). Genome sequences utilized in our analyses are listed with their accession numbers in Table S1.

### Prediction of plasmids and prophage

RAST annotations were searched for the following terms to identify putative plasmids and prophage: plasmid, par, phage, replication. Each contig containing a gene annotated with one of these terms was visually inspected for additional gene content indicative of plasmid or phage origin. Contigs predominantly composed of such genes were deemed putative plasmids/prophage. Additionally, the annotations of each contig under 60 kb in length were visually examined to identify putative plasmids/prophage based on gene content. Finally, the genomes were searched by blastn using plasmid sequences of close relatives as queries.

### Phylogenomic analysis

Genes that were most similar in fly isolates were identified as sharing terminal nodes of amino acid sequence-based trees (see below for specific comparisons). The first round of analysis was performed by building a nearest neighbor tree from an alignment of all amino acid sequences for a given COG using Muscle (Edgar, 2004). A comprehensive set of all terminal nodes in each tree was derived using the R package “ape” (Paradis et al., 2004), any tree with a node occupied by only the taxa in one of the following groupings was selected for additional analysis: *A. pomorum*^F^ and *A. tropicalis*^F^; *L. brevis*^F^, *L. fructivorans*^F^, and *L. plantarum*^F^; *L. brevis*^F^ and *L. brevis* EW; and *L. plantarum*^F^ and *L. plantarum* WJL. For each COG identified in the first round analysis, maximum likelihood trees were built using default parameters and at least 1000 bootstraps using PUZZLE-TREE. Trees with branch support of 80% or greater were retained in the final list.

## Results

### Phenotypic traits of gut microbiota isolates

As part of our preliminary characterization of bacteria isolated from the *Drosophila* gut microbiota, we tested their carbon utilization and chemical resistance traits (Table S2). For *A. pomorum*^F^ the highest metabolic activity was obtained with glycyl-L-proline or glucuronamide as the sole carbon source, while dextrin, glucose, galactose and acetoacetic acid also produced positive tests (defined as absorbance increase ≥15% above the negative control; Figure S1A). *A. tropicalis*^F^ showed a high level of metabolic activity when mannose, sucrose, glycerol, methyl pyruvate, lactic acid or formic acid was provided. Both *Acetobacter* species were resistant to lincomycin, vancomycin, nalidixic acid, and aztreonam, though it is important to note this may have been due to acid produced by the bacteria degrading pH sensitive antibiotics (Figure S1B). In subsequent analyses of the genomic content of *A. pomorum*^F^ and *A. tropicalis*^F^ (described below) we found genetic evidence supporting 68% (38/56) of the positive carbon source utilization reactions.

The carbon utilization patterns of *L. brevis*^F^ and *L. plantarum*^F^ were very different from one another. While both strains tested positive for glucose, dextrin, maltose, and fructose, *L. plantarum^F^* could also utilize a range of sugars and sugar alcohols that *L. brevis*^F^ could not (Figure S2). These included lactose, mannose, galactose, melibiose, trehalose, cellobiose, gentibiose, sucrose, turanose, sorbitol, and mannitol. *L. brevis*^F^ could utilize a number of compounds that *L. plantarum*^F^ could not, including galactonic acid, glucuronic acid, glucuronamide, acetoacetic acid and inosine (Figure S2). The Lactobacilli were sensitive to most of the chemical stressors tested, but both showed resistance to vancomycin, nalidixic acid, aztreonam and potassium tellurite. *L. brevis*^F^ was resistant to rifamycin SV, while *L. plantarum*^F^ was slightly sensitive. Subsequent genomic analyses (described below) found genetic evidence supporting 74% (29/39) of the positive carbon source utilization assays.

### Impact of microbiota of fly and non-fly origins on *Drosophila traits*

The availability of bacteria closely related to the *Drosophila* microbiota, but isolated from non-fly environments, provided us with the opportunity to assess whether fly-associated bacteria are functionally distinct from their non-fly counterparts. We reared gnotobiotic *Drosophila* with gut microbiota consisting of single bacterial species as well as 5-species communities comprising all fly (^F^) or non-fly (^NF^) isolates, and compared the effect of these treatments on gut microbiota-responsive host traits. These comparisons were between conspecific isolates for all species except *A. pomorum*^F^, which was compared to a non-fly isolate of the closely related species *A. pasteurianus*.

**Figure 1:**
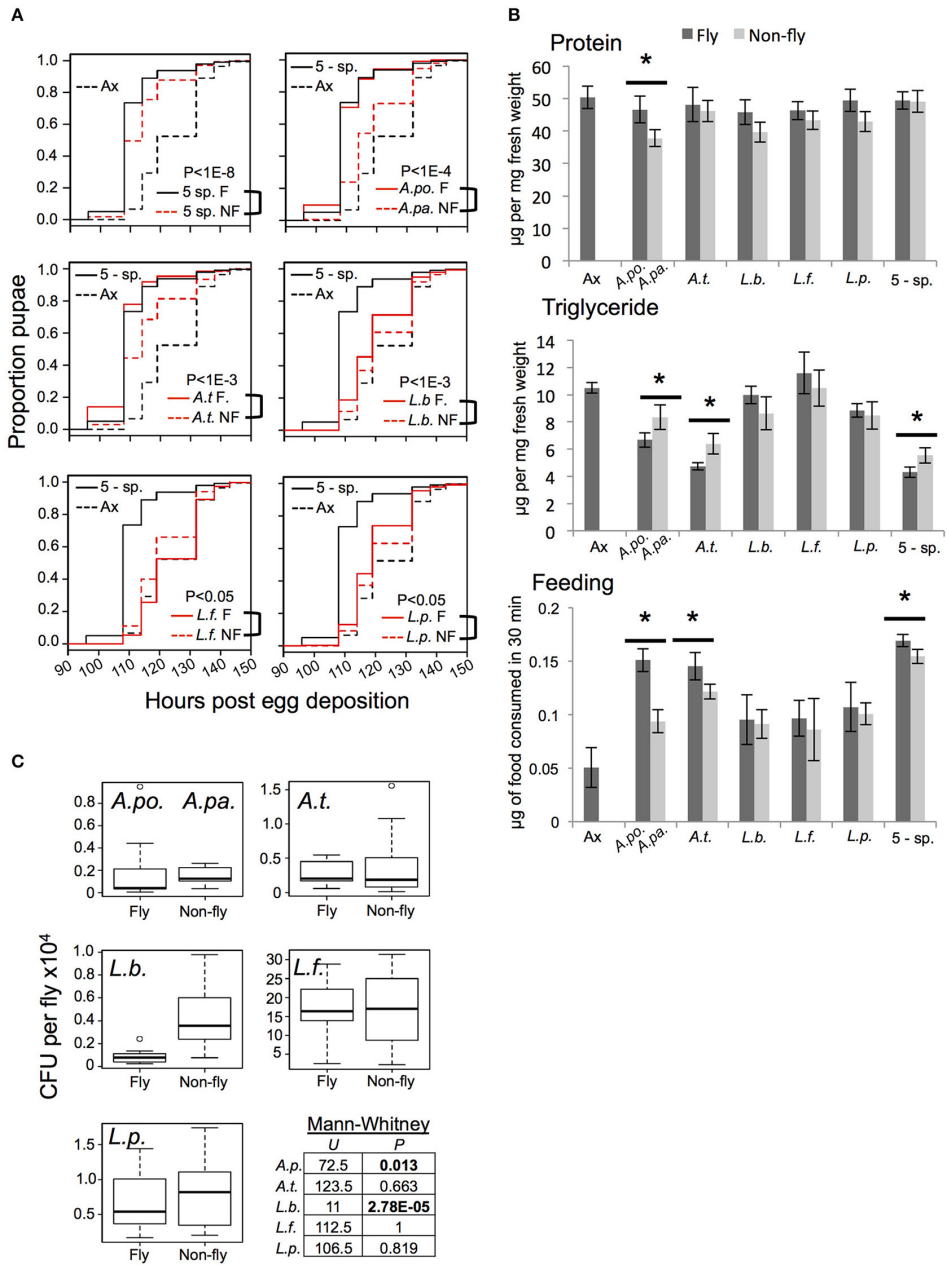
**Functional comparison of gnotobiotic *Drosophila* colonized by bacteria of fly origin (^F^) and non-fly origin (^NF^) (A) Kaplan-Mayer plots comparing the larval development time of gnotobiotic flies reared with the indicated microbiota treatments**. Each plot includes data from axenic (dotted black line) and 5-species gnotobiotic (solid black line) single fly microbiota species gnotobiotic (solid red line) and single non-fly (NF) bacteria-associated flies (dotted red line) except for the top left panel in which the dotted red line represents the 5-species NF gnotobiotic treatment. *P*-value indicates the result of mixed effects cox model comparison of the bracketed strains. **(B)** Flies raised with the microbiota treatments indicated were compared for protein and triglyceride content, and food consumption. Statistically significant differences by pairwise *t*-test are indicated by ^*^ for *P* < 0.05 after correction for multiple comparisons; data presented are mean ± SE from 4 to 5 biological replicates, each with 3–5 technical replicates. Dark bars indicate axenic or fly isolate treatments, while light bars indicate non-fly isolate treatments. There were not significant changes in host glucose content between microbiota treatments (not shown). **(C)** Bacterial abundance was assessed by homogenization and plating of whole flies. A.po., *A. pomorum*; A.pa. *A. pasteurianus* (^NF^); A.t., *A. tropicalis*; L.b., *L. brevis*; L.f., *L. fructivorans*; L.p., *L. plantarum*; 5-sp., all 5 species.

Consistent with prior studies (Bakula, 1969; Shin et al., 2011; Storelli et al., 2011; Ridley et al., 2012; Newell and Douglas, 2014; Wong et al., 2014), we found that axenic hosts displayed prolonged larval development time, decreased food consumption and altered nutrient profile, including a heightened triglyceride level, relative to gnotobiotic flies with a 5-species^F^ microbiota (Figures 1A,B). The 5-species^NF^ community partially restored gut microbiota-responsive traits, but showed a significantly increased development time, and elevated triglyceride level compared to the 5-species^F^ community of fly origin (Figures 1A,B). Flies with the 5-species^NF^ microbiota also showed a small but significant reduction in food consumption compared to the 5-species^F^ microbiota.

Flies colonized by individual species of bacteria displayed distinct phenotypes. Generally, fly and non-fly isolates of the same species had qualitatively similar affects on host traits, but there were some statistically significant differences. Most notably, *A. pasteurianus*^NF^ and *A. tropicalis*^NF^ conferred slower development, higher triglyceride levels, and decreased food consumption compared to the related isolates of fly origin (*A. pomorum^F^* and *A. tropicalis^F^*, respectively; Figures 1A,B). Additionally, *A. pasteurianus*^NF^ mono-colonized flies had significantly reduced protein content compared to flies bearing *A. pomorum*^F^ (Figure 1B). The impact of Lactobacilli on development was varied: *L. brevis*^NF^ and *L. plantarum*^NF^ prolonged larval development compared to fly isolates, while *L. fructivorans*^NF^ significantly reduced development time compared to *L. fructivorans*^F^, which was the only bacterial treatment that did not significantly reduce development time compared to axenic flies (Figure 1A). To check whether the bacteria tested were capable of proliferating in the host environment, bacterial abundance in whole flies was determined. The non-fly isolates reached levels equivalent to or significantly greater than the level of the corresponding strain isolated from the fly gut (Figure 1C).

These functional comparisons of microbiota from fly vs. non-fly origins reveal quantitative differences in microbiota-responsive host phenotypes. The results suggest that genetic differences between these closely related, but functionally distinct, bacteria may include strain-specific adaptations to the gut microbiota niche. Identification and characterization of these differences could help elucidate the basis for microbiota functions.

### The genome sequences of gut microbiota isolates

For each of the five gut microbiota isolates, we determined a draft genome sequence by short read sequencing and de novo assembly. The draft assemblies generated have predicted sizes, and %GC content characteristic of their respective species (Table 2). Final draft assemblies, outlined in Table 2, were submitted to the RAST server for automated annotation (Aziz et al., 2008). To compare orthologous gene content, we implemented orthoMCL to generate clusters of orthologous groups (COGs) from a panel of related genome sequences. This identified unique gene content in fly vs. non-fly strains used in this study (Table 3, Tables S3–S7).

**Table 2:** **Genome assembly information**.

**Species**	**Total bp**	**Features**	**rrna operons**	**trna genes**	**% GC**	**Contigs**	**Max size (bp)**	**N50 (bp)**
*A. pomorum*^F^	2,845,508	2840	1	45	52.4	137	466,505	90,072
*A. tropicalis*^F^	3,747,493	3654	1	44	55.5	129	273,701	116,787
*L. brevis*^F^	2,871,827	2866	3	69	45.4	117	683,327	290,627
*L. fructivorans*^F^	1,333,965	1350	2	62	39.2	38	357,852	174,419
*L. plantarum*^F^	3,195,557	3131	1	60	44.5	88	282,956	124,826

**Table 3:** **Shared and unique gene content between fly and non-fly isolates**.

**A vs. B**	**Shared**	**Unique to A**	**Unique to B**
*A. pomorum*^F^ vs. *A. pasteurianus*^NF^	2215	577	685
*A. pomorum*^F^ vs. *A. pomorum* DM001 (fly isolate)	2250	542	150
*A. tropicalis*^F^ vs. *A. tropicalis*^NF^	2617	1037	941
*L. brevis*^F^ vs. *L. brevis*^NF^	1492	1197	1371
*L. brevis*^F^ vs. *L brevis* EW (fly isolate)	2498	291	314
*L. plantarum*^F^ vs. *L. plantarum*^NF^	2641	425	390
*L. plantarum*^F^ vs. *L. plantarum* WJL (fly isolate)	2846	220	512
*L. fructivorans*^F^ vs. *L. fructivorans*^NF^	1206	75	132

### Whole genome alignments

Genome-wide nucleotide alignments were performed to assess the relatedness of fly isolates to other publicly available genome sequences. This analysis revealed an Average Percent Nucleotide Identity (APNI) of >99.9% between our *A. pomorum*^F^ isolate and *A. pomorum* DM001, which is also a *Drosophila* microbiota isolate (Shin et al., 2011). This clearly differentiates *A. pomorum* from *A. pasteurianus* representatives, as these genomes have ~90% APNI which is below 95% APNI considered to be a benchmark species-level cutoff (Table 4; Richter and Rossello-Mora, 2009). The *A. tropicalis*^F^ isolate shows 93.3% APNI with the genome of *A. tropicalis*^NF^ (Table 4), and APNI <87% with all other publicly available *Acetobacteraceae* genomes (data not shown). We have provisionally assigned *A. tropicalis*^F^ its species designation based on this observation, and the fact that its 16S rRNA gene sequence is >99% identical to that of *A. tropicalis*^NF^.

**Table 4:** **Whole genome alignment comparisons of *Acetobacter* species**.

**Average percent nucleotide identity (APNI)**							
***Acetobacter***	***tropicalis*^F^**	***tropicalis* NBRC 101654**	***pomorum*^F^**	***pasteurianus* NBRC 101655**	***pasteurianus* NBRC 106471**	***pomorum* DM001**	***pasteurianus* 3p3**
*pasteurianus* 386B	85.2	84.7	90.6	99.4	97.6	90.6	92.2
*pasteurianus* 3p3	84.9	84.6	90.9	92.4	92.5	91.1	
***pomorum* DM001**	85.8	84.5	**99.9**	90.6	90.8		
*pasteurianus* NBRC 106471	86.0	84.8	90.6	97.7			
*pasteurianus* NBRC 101655	85.0	84.6	90.6				
***pomorum*^F^**	86.9	84.3					
*tropicalis* NBRC 101654	**93.2**						

*Abbreviations in bold indicate fly isolates, highest value for each fly isolate is bolded*.

Whole genome APNI between *L. brevis*^F^ and *L. brevis*^NF^ is quite low (91.32%), but much higher with the human feces isolate *L. brevis* ATCC 14869 (>99%) and a recently sequenced fly gut isolate *L. brevis* EW (98.7%; Table 5). The *L. plantarum*^F^ genome has APNI >98.9% with all other *L. plantarum* genomes tested, showing the highest value in comparison with strain NC8 (>99.9%). The latter observation is interesting because NC8 is a silage isolate known to be free of plasmids, a rare condition for *L. plantarum*. Using several search strategies, we found no evidence for plasmids in the draft genome of our isolate (see Materials and Methods). *L. fructivorans*^F^ shares 97.3% APNI with *L. fructivorans*^NF^ (Table 5).

**Table 5:** **Whole genome alignment comparisons of *Lactobacillus* species**.

**Average percent nucleotide identity (APNI)**							
***Lactobacillus plantarum* strains**	**WJL**	**WCFS1**	**ST-III**	**P-8**	**NC8**	**IPLA88**	***plantarum*^F^**
ZJ316	98.8	98.9	98.9	99.1	98.9	99.2	98.9
***plantarum*^F^**	99.3	99.1	99.3	98.9	**99.9**	99.1	99.0
IPLA88	99.1	99.3	99.1	98.9	99.1		
NC8	99.2	99.1	99.3	98.9			
P-8	98.7	98.9	98.9				
ST-III	**99.6**	99.1					
WCFS1	98.9						
***Lactobacillus* species**	***fructivorans*^F^**	***fructivorans* KCTC 3543**	***brevis*^F^**	***brevis* KB290**	***brevis* EW**	***brevis* ATCC 27305**	
*brevis* ATCC 14869	89.4	87.6	**99.0**	97.7	**99.0**	88.6	
*brevis* ATCC 27305	87.0	85.3	91.3	92.2	90.0		
***brevis* EW**	89.8	88.8	98.7	97.6			
*brevis* KB290	88.7	87.4	97.5				
***brevis*^F^**	89.7	88.3					
*fructivorans* KCTC 3543	**97.3**						

*Abbreviations in bold indicate fly isolates, highest value for each fly isolate is bolded*.

### Metabolic pathway predictions for fly isolates

To begin analyzing the genomic content of the fly gut microbiota isolates, we first examined the metabolic pathways predicted for each bacterium utilizing tools in RAST and extensive manual curation (e.g., BLASTp searches against the genomes to check for mis-annotated gaps in metabolic pathways). *A. tropicalis*^F^ is capable of de novo synthesis of all amino acids, B vitamins, porphyrin and terpenoid backbones (Table S8). *A. pomorum*^F^ shares most of these functions, but is missing a PdxB homolog for synthesis of vitamin B6, and several genes for the production of porphyrin and terpenoid backbones (Table S8). *A. pomorum^F^* also appears to have a reduced capacity for fatty acid degradation/modification, as it lacks an acyl-CoA dehydrogenase homolog. Investigating the genome of *A. pomorum* DM001, we found that it also lacks acyl-CoA dehydrogenase and PdxB homologs.

Past analyses of *Lactobacillus* genomes have found them to be typified by diverse repertoires of sugar utilization systems and incomplete biosynthetic capacity for a number of amino acids and vitamins (Makarova et al., 2006; Capozzi et al., 2012). Various studies have suggested B vitamin or amino acid provisioning could be important functions of the *Drosophila* gut microbiota (Blatch et al., 2010; Storelli et al., 2011; Fridmann-Sirkis et al., 2014; Wong et al., 2014). Based on our genomic data, *L. plantarum*^F^ is capable of synthesizing all but the branched chain amino acids, while *L. brevis*^F^ can produce only alanine, aspartate, glutamate, asparagine and glutamine. *L. fructivorans*^F^ lacks key steps in all amino acid biosynthetic pathways (Table S8). *L. plantarum*^F^ can produce riboflavin, nicotinamide, and folate, but no other B vitamins. *L. brevis*^F^ can synthesize riboflavin and nicotinamide, while *L. fructivorans*^F^ cannot complete biosynthesis of any B vitamins (Table S8). We extended our analysis by searching the genomes of fly isolates *L. brevis* EW and *L. plantarum* WJL (Kim et al., 2013a,b). Here we found that *L. plantarum* WJL has the same amino acid production capacity as our *L. plantarum*^F^ isolate, and genes for producing riboflavin and folate but not nicotinamide. *L. brevis* EW resembles *L. brevis*^F^ in terms of amino acid production ability, but cannot complete synthesis of any B vitamins.

Annotation searches uncovered a wide array of sugar utilization pathways in *L. plantarum*^F^ including catabolic enzymes for 12 mono- and disaccharides, as well as sugar phosphotransferase systems (PTS) predicted for transport of 15 different sugars and sugar alcohols (Table S9). By contrast *L. brevis*^F^ possesses just 4 PTS systems, and catabolic enzymes for 11 sugars. Examining the carbon source utilization abilities of *L. brevis*^F^ and *L. plantarum*^F^ (Figure S2), we saw strong agreement between the genomic predictions and observed utilization patterns. Lastly, *L. fructivorans*^F^ has a single PTS system predicted to import glucose, and catabolic enzymes for just 5 sugars: glucose, fructose, xylose, lactose, and galactose (Table S9).

### Gene content comparisons between fly and non-fly isolates

Next we compared the gene content of the fly and non-fly strains characterized in this study to gain insight into the predicted functions that may differ between these bacteria. Recognizing that a single two-way comparison of genomes has limited power, we cross-referenced these comparisons with other genome-sequenced members of the species wherever possible. One way this was achieved was by using BLASTp to check whether genes unique to a fly isolate in a two-way comparison were shared among other fly isolates. In the first two-way comparison, we found 1037 genes unique to *A. tropicalis*^F^ compared to *A. tropicalis*^NF^, about half of which encode hypothetical proteins. Annotated unique genes included a large number associated with mobile elements and detoxification systems (Table S4). Putative mobile element genes include three prophage, several predicted plasmid replication loci, Tra and Trb family conjugative transfer genes, and a CRISPR locus with seven CAS genes. Detoxification systems include dehalogenation enzymes, aromatic alcohol and aldehyde degradation, superoxide dismutase, organic hydroperoxide resistance, and three heavy metal efflux systems. Metabolic functions predicted for *A. tropicalis*^F^ but absent from *A. tropicalis*^NF^ include urea degradation, ethanolamine utilization, and the potential to use fumarate as an electron acceptor.

There are 577 genes in *A. pomorum*^F^ that are absent from *A. pasteurianus*^NF^. This list includes genes encoding a glycogen de-branching enzyme, a capsular polysaccharide synthesis locus, and several iron scavenging (ferrichrome and siderophore) receptors, (Table S3). In this list we also found several groups of orthologs in common with *A. tropicalis*^F^, including two predicted prophage, all seven CAS genes and the Tra/Trb conjugative transfer genes Putative mobile genes shared between the fly gut microbiota isolates could suggest that there has been recent genetic exchange between these isolates, consistent with their shared environment. A list of predicted plasmids, phage, and CRISPR associated proteins from the genomes sequenced for this study is displayed in Supplementary Material (Table S10).

Both *L. fructivorans*^F^ to *L. fructivorans*^NF^ have a relatively small genome size (less than 1.4 Mb). The 75 genes unique to *L. fructivorans*^F^ include a number of interesting predicted functions: five transporters, two transcriptional regulators, two transposases, two phage or prophage proteins, a large LPXTG-motif surface protein, and a CRISPR region with five CAS proteins. Top BLAST hits from the CAS proteins suggest that this locus is homologus to CRISPR loci in a number of other Lactobacilli, and belongs to the Csn1-type CRISPR family (Horvath et al., 2009).

Pairwise comparisons of gene content in fly vs. non-fly *L. plantarum* strains revealed ~400 genes unique to each strain. *L. plantarum*^F^ is distinguished from *L. plantarum*^NF^ by predicted PTS systems for fructose and mannose import, 12 cell surface proteins, and two extracellular polysaccharide synthesis loci. Unlike our isolate, *L. plantarum*^NF^ lacks the capacity synthesize riboflavin due to the absences of RibC and RibD homologs (but can produce nicotinamide and folate). Of the features we found unique to *L. plantarum*^F^ relative to *L. plantarum*^NF^, the PTS systems, polysaccharide synthesis loci, and 10 of the 12 surface proteins were present in *L. plantarum* WJL. In fact, *L. plantarum*^F^ shared more protein coding genes with *L. plantarum* WJL (2846) than with any other *L. plantarum* strain analyzed, and the two fly isolates were more similar by this metric than were any other pair of *L. plantarum* strains (Table S11).

The genomes of *L. brevis*^F^ and *L. brevis*^NF^ are very divergent, in part because the non-fly strain has an unusually large genome compared to other sequenced *L. brevis* isolates (3.14 Mb vs. 2.32–2.8 Mb). More than 1300 genes present in *L. brevis*^NF^ are absent from the fly isolate, including genes predicted to enable de novo biosynthesis of purines and 16 amino acids, which are absent from *L. brevis*^F^ (Table S5). Despite a broader capacity for amino acid production, *L. brevis*^NF^ is missing the genes for riboflavin synthesis. Notable predicted metabolic capabilities unique to *L. brevis*^F^ include genes for the import of citrate and its degradation to oxaloacetate by citrate lyase. Also, *L. brevis*^F^ has a 21 gene locus for the production and/or utilization of propanediol, a capacity known to be present in some other Lactobacilli (Khan et al., 2013). The genome of fly isolate *L. brevis* EW contains orthologs for both the citrate and propanediol pathways. As with the *L. plantarum* fly isolates, the two *L. brevis* fly isolates shared more genes (2498) than any other pair of *L. brevis* strains (Table S11).

### All vs. all comparisons of fly vs. non-fly isolate gene content

We next asked the question, what genes are shared among all *Acetobacteraceae* fly isolate genomes but absent from those of non-fly isolates? Examining the clusters generated by orthoMCL, we found that no genes fit these criteria. Confining the analysis to *Acetobacter* fly isolates (*A. malorum* DmCs_005, *A. tropicalis* DmCs_006, *A. pomorum* DmCs_004 and DM001) we identified three shared genes that are absent from non-fly isolates. The predicted functions of these genes suggest they encode homologs of MobACD, and are part of a plasmid partitioning/transfer locus (Table S12). Further examination of the genomic context of the genes in each *Acetobacter* genome did not suggest that they are part of a functioning plasmid, however. The genes were found in a unique context in each of the genomes, adjacent to different genes and on large contigs that do not resemble plasmids (data not shown). A similar comparison of fly vs. non-fly *Lactobacilli* found that no genes are shared among fly isolated *Lactobacilli* but absent from all other *Lactobacillus* genomes. Taken together, these results indicate that the *Drosophila*-associated bacteria we analyzed cannot be differentiated from their “free-living” relatives based solely on gene content.

### Phylogenomic inference of shared gene function in microbiota genomes

**Figure 2:**
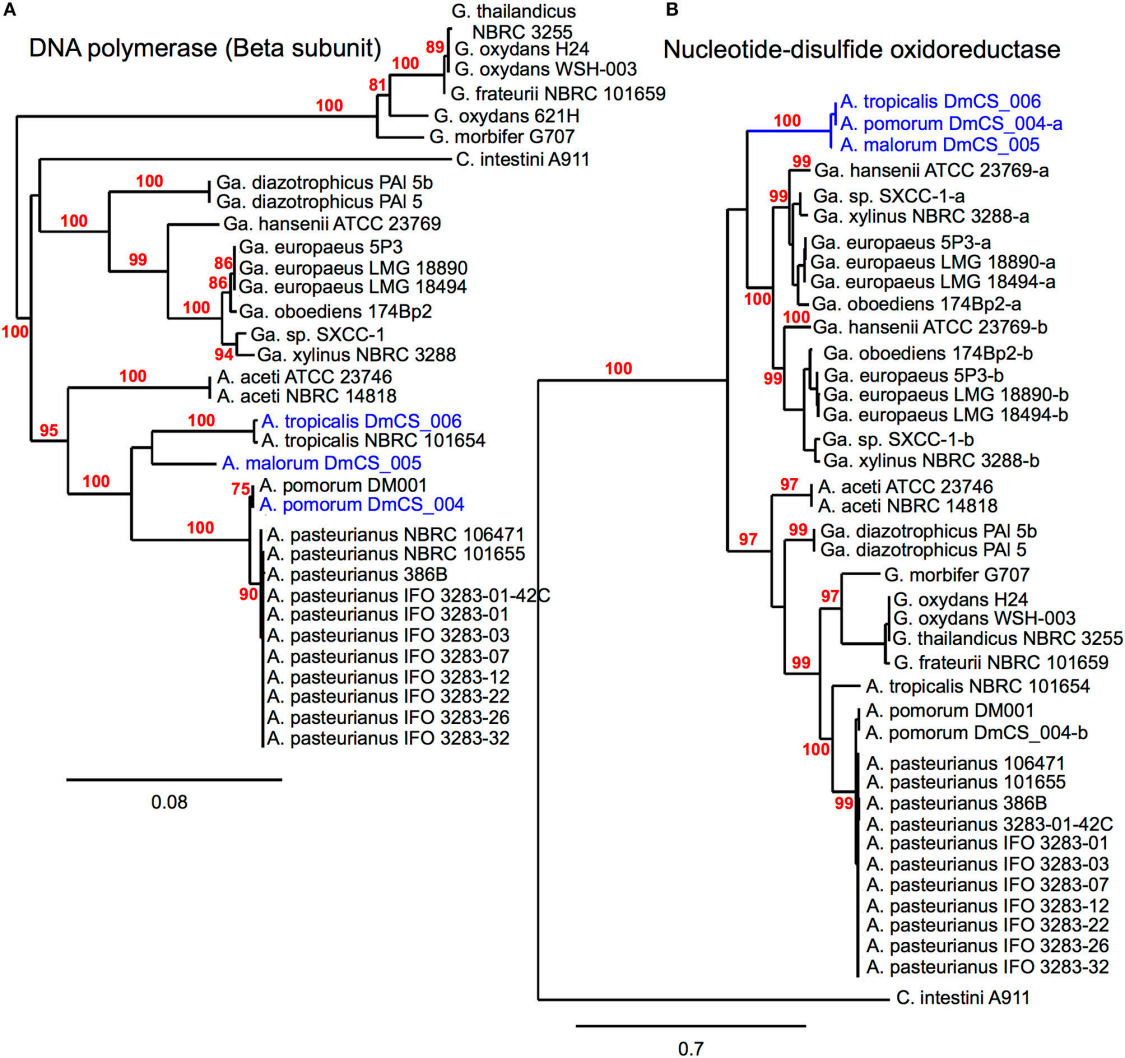
**Phylogenomic inference of shared functions among microbiota genes**. This figure depicts two protein sequence trees, **(A)** in which *Acetobacter* fly isolates cluster with their conspecific non-fly counterparts and **(B)** in which our fly isolates (blue) share a terminal node, meeting our selection criteria. **(A)** compares sequences from a DNA polymerase subunit, while **(B)** compares sequences for a pyridine nucleotide-disulfide oxidoreductase, present in the 2-alkenal reductase locus depicted in Figure 3E. In cases where more than one sequence was present within a genome, a dash and lowercase letter are added after the strain designation to differentiate between them. Bootstrap support values are displayed in red for branches with ≥75% confidence after 1000 bootstraps.

Unique gene content can be one indicator of niche specialization in symbiotic bacteria relative to free-living ones. However, it is also possible for a gene family present in both symbiotic and non-symbiotic bacteria to functionally diverge due to selective pressures unique to their environment. Phylogenomic inference, which utilizes phylogenetic comparisons to inform predictions of gene function, has been used successfully to pinpoint distinct functions in sub families of homologous genes (Eisen, 1998; Brown and Sjolander, 2006). We reasoned that protein coding genes for which fly isolate representatives form a monophyletic clade might have shared functions that are relevant for survival in the gut microbiota niche (Figure 2). This analysis was aided by the fact that many orthologous genes in the *A. pomorum*^F^ and *A. tropicalis*^F^ genomes are more closely related to non-fly relatives than they are to each other. To identify orthologs from fly isolates that are monophyletic, we built phylogenetic trees for amino acid sequences from each of 8283 COGs that are shared in the genomes of 33 *Acetobacteraceae* strains (including the genera *Commensalibacter, Acetobacter, Gluconobacter, and Gluconacetobacter*). For 15 COGs, sequences from *A. tropicalis*^F^ and *A. pomorum*^F^ formed a terminal node, suggesting that the gene products in these taxa may have a more closely shared function than with other members of the *Acetobacteraceae* (Table 6). The phylogenetic relationship of these sequences was validated by maximum likelihood analysis, in which >80% of bootstraps supported their assignment to the same terminal node. For comparison, only 3 sequences met these criteria when comparing *A. tropicalis*^NF^ and *A. pasteurianus*^NF^ (data not shown).

**Table 6:** **Genes more closely related among gut *Acetobacter* isolates than to orthologs in non-fly isolates**.

**Locus^∧^**	**Predicted function**	**# taxa with # COG (of 33)**	**Fly isolates with COG**	**Bootstrap confidence (%)**
A	FAD-dependent pyridine nucleotide-disulfide oxidoreductase	33^*^	*A. pomorum^F^, A. tropicalis^F^, A. malorum^F^*	100
			*A. pomorum* DM001, *C. intestini* A911	
A	Aldehyde dehydrogenase	3	*A. pomorum^F^, A. tropicalis^F^, A. malorum^F^*	n/a^$^
A	Transcriptional regulator TetR-like	3	*A. pomorum^F^, A. tropicalis^F^, A. malorum^F^*	n/a
A	2-alkenal reductase	8	*A. pomorum^F^, A. tropicalis^F^, A. malorum^F^*	100
A	Aldehyde dehydrogenase	25^*^	*A. pomorum^F^, A. tropicalis^F^, A. malorum^F^*	97
A	Protease	14	*A. pomorum^F^, A. tropicalis^F^, A. malorum^F^*	100
A	Transcriptional regulator, TetR-like	17^*^	*A. pomorum^F^, A. tropicalis^F^, A. malorum^F^*	100
B	Flavoprotein wrbA-like	18	*A. pomorum^F^, A. tropicalis^F^, A. malorum^F^,C. intestini* A911	100^#^
B	RND efflux system, fusion protein	4	*A. pomorum^F^, A. tropicalis^F^, A. malorum^F^*	100^#^
B	RND efflux system, lipoprotein	9^*^	*A. pomorum^F^, A. tropicalis^F^, A. malorum^F^*	100^#^
			*A. pomorum DM001, C. intestini* A911	
B	LysR-like transcriptional regulator	9	*A. pomorum^F^, A. tropicalis^F^, A. malorum^F^*	100^#^
Lone	Aldehyde dehydrogenase	25^*^	*A. pomorum^F^, A. tropicalis^F^, A. malorum^F^*	97
			*A. pomorum DM001, C. intestini* A911	
Lone	Hypothetical protein, phage locus	3^*^	*A. pomorum^F^, A. tropicalis^F^, A. malorum^F^*	n/a
Lone	Hypothetical protein, mobDE locus	3	*A. pomorum^F^, A. tropicalis^F^, A. malorum^F^*	n/a
Lone	FAD-dependent pyridine nucleotide-disulfide oxidoreductase	33^*^	*A. pomorum^F^, A. tropicalis^F^, A. malorum^F^*	100
			*A. pomorum DM001, C. intestini* A911	
Lone	Transcriptional regulator, TetR-like	17^*^	*A. pomorum^F^, A. tropicalis^F^, A. malorum^F^*	100
Lone	RND efflux system, lipoprotein	9^*^	*A. pomorum^F^, A. tropicalis^F^, A. malorum^F^*	100^#^
			*A. pomorum DM001, C. intestini* A911	
Lone	Hypothetical protein, phage locus	3^*^	*A. pomorum^F^, A. tropicalis^F^, A. malorum^F^*	n/a

∧ *Genes in the same genomic locus share a letter (e.g., A) or are in unique “lone” loci*.

* *Some taxa contain more than one copy of this gene*.

# *In these cases, A. pomorum^F^, A. tropicalis^F^, and A. malorum^F^ sequences form a terminal node with a highly similar sequence from G. xylinus NBRC 3288*.

$ *n/a indicates that the only genomes with this COG are A. pomorum^F^, A. tropicalis^F^, and A. malorum^F^*.

**Figure 3:**
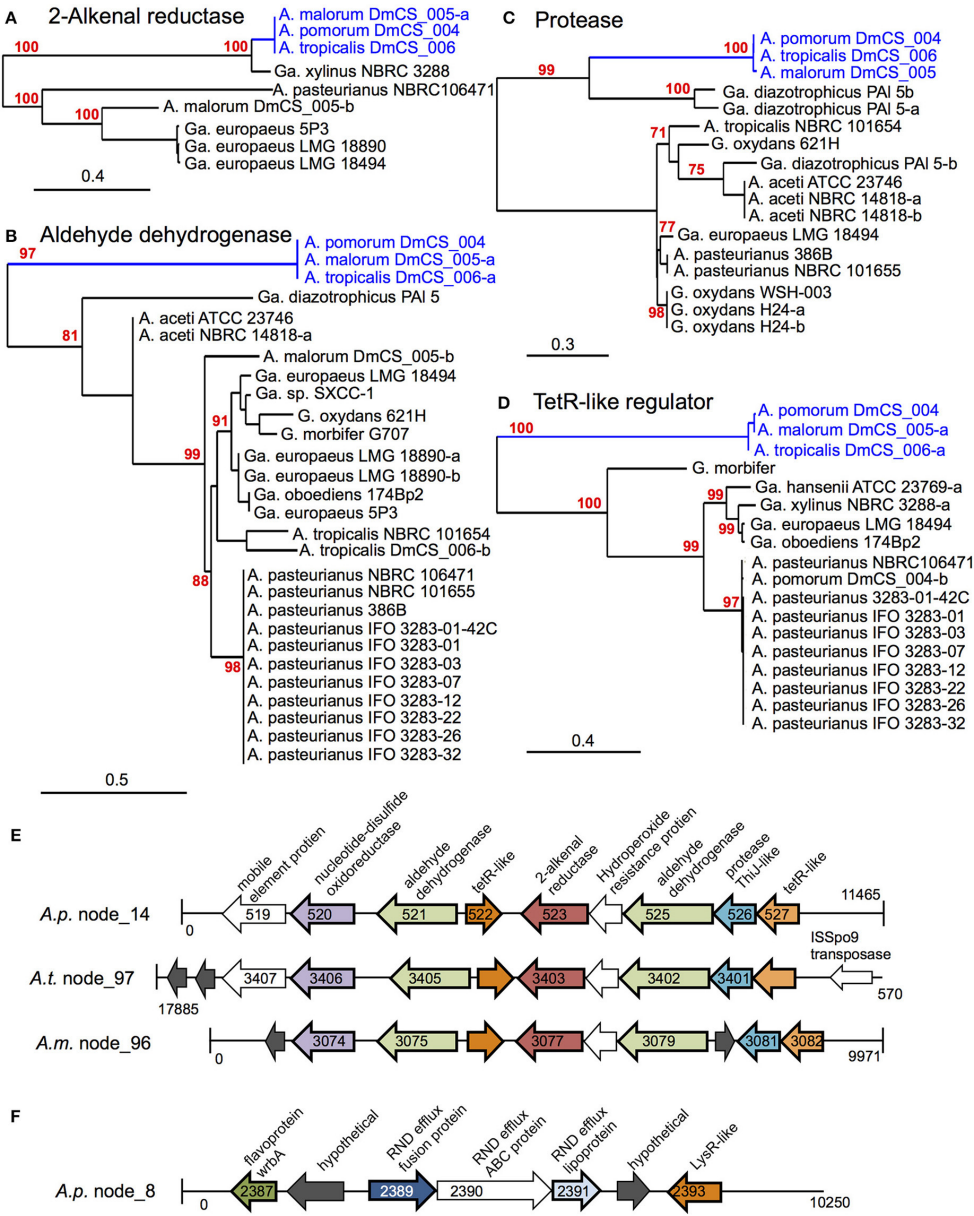
**Genomic loci identified by phylogenomic analysis. (A–D)** Maximum likelihood trees comparing protein sequences of several genes identified in the 2-alkenal reductase locus depicted in **(E)**. In cases where more than one sequence was present within a genome, a dash and lowercase letter are added after the strain designation to differentiate between them. Bootstrap support values are displayed in red for branches with ≥75% confidence after 1000 bootstraps. **(E)** The 2-alkenal reductase locus from *A. pomorum*^F^ (*A.p*.), *A. tropicalis*^F^ (*A.t*.), and *A. malorum*^F^ (*A.m*.), which contain orthologous genes identified by phylogenomic analysis. Organism and contig (node) numbers are indicated on the left, base pair numbers are indicated at the start and end of each linear DNA depiction with vertical lines indicating the end of a contig. Gene annotations at the top apply to all like-colored genes below, genes identified by phylogenomic analysis appear in color with dark outline, while other annotated genes appear in white and hypothetical genes appear in dark gray. Peg numbers are indicated on top of gene arrows where space allowed. **(F)** Diagram of the Cme-like efflux locus in the genome of *A. pomorum*^F^ (*A.p*.). Loci in *A. tropicalis*^F^ and *A. malorum*^F^ genomes show the same synteny (not shown).

A total of 18 protein sequences were represented by the 15 COGs, including two gene products predicted to bind lipid, 3 aldehyde dehydrogenase sequences, and 5 gene products involved in oxidation/ reduction reactions. Eight of the 18 are encoded in a 10-gene locus in *A. tropicalis*^F^ and *A. pomorum*^F^ (Figures 3A–E). This locus encodes an FAD-dependent NADH dehydrogenase, two aldehyde dehydrogenases, two transcriptional regulators, a 2-alkenal reductase, a ThiJ-like protease, an OsmC-like organic hydroperoxide resistance protein. It shows perfect synteny, between the two genomes, and is flanked by mobile element protein sequences, suggesting that it may be a mobilizable genetic locus that may have been recently acquired horizontally.

Of the remaining 11 proteins for which *A. tropicalis*^F^ and *A. pomorum*^F^ share a terminal node, 4 are encoded by a second genomic locus (Figure 3F), including components of an efflux transporter similar to the *Campylobacter* multi-drug efflux system CmeABC. In *Campylobacter jejuni*, the CmeABC transporter has been characterized as a broad-specificity efflux pump conferring resistance to several antibiotics, heavy metals, bile salts, and other antimicrobial agents (Lin et al., 2002). The final 7 were located on unique contigs. Further, when we sequenced these loci in *A. malorum* DmCS_005, another *Acetobacter* isolate from *Drosophila*, we found that the sequences of all 18 genes were most similar to the other two fly isolates (*A. tropicalis*^F^ and *A. pomorum*^F^), i.e., the three isolates shared a terminal node for each of the 18 identified genes. In summary, this approach identified two genetic loci that function in oxidation/reduction, aldehyde dehydrogenase activity, and detoxification, which we predict to have a shared function in *Acetobacter* species inhabiting the *Drosophila* gut.

To identify *Lactobacillus* genes that may be adapted for the fly gut microbiota niche, we performed the same phylogenomic analysis described above. When taking into account 10,097 COGs shared among 80 *Lactobacillus* genomes, we found no instance where sequences from all five fly isolates shared a terminal node. This could be due to a number of factors, including the lower overall sequence conservation between these isolates (Table 5). To circumvent this problem, we next asked if there were instances where the conspecific pairs of fly isolates (i.e., *L. plantarum* WJL and *L. plantarum*^F^; *L. brevis* EW and *L. brevis*^F^) share a terminal node on orthologous protein trees. For *L. brevis*, 541 genes met this criterion (Table S13) indicating a high degree of shared gene phylogeny between the two genomes, but limiting our ability to make conclusions about shared functions specific to symbiosis.

Phylogenomic analysis of *Lactobacillus* orthologs uncovered 12 instances where proteins from *L. plantarum*^F^ and *L. plantarum* WJL share a terminal node (Table 7). Of these, 5 were hypothetical proteins that were only present in these two taxa. The remaining 7 included a nucleoside-diphosphate-sugar epimerase, a muramidase, a predicted acyl-transferase, and an RNA methyltransferase. The diphosphate-sugar epimerase is predicted to function in the synthesis of extracellular polysaccharides; these molecules may contribute to biofilm formation or binding to host surfaces. Unlike the *Acetobacter* genes identified by our phylogenomic approach, the *L. plantarum* genes were not concentrated at particular loci but distributed throughout the genome.

**Table 7:** **Genes more closely related among two gut *L. plantarum* isolates than to orthologs in other taxa**.

***L.p*.^F^ peg No**.	**Annotation**	**No. of taxa with gene**	**Fly isolates with COG**	**Bootstrap confidence (%)**
2404	Nucleoside-diphosphate-sugar epimerase	44	*L.p*.^F^ *L.p*.^WJL^ *L.b*.^F^ *L.b*.^EW^	87
2415	RNA methyltransferase, TrmA family	80^*^	all	97
693	Mobile element protein	3	*L.p*.^F^ *L.p*.^WJL^	100
2668	Acyl-transferase 3 superfamily	19	*L.p*.^F^ *L.p*.^WJL^	82
890	Hypothetical protein	80^*^	all	90
2477	Muramidase	5	*L.p*.^F^ *L.p*.^WJL^	100
3023	Hypothetical protein	4	*L.p*.^F^ *L.p*.^WJL^	100
1699	Hypothetical protein	2	*L.p*.^F^ *L.p*.^WJL^	n/a^∧^
2082	Hypothetical protein	2	*L.p*.^F^ *L.p*.^WJL^	n/a
370	Hypothetical protein	2	*L.p*.^F^ *L.p*.^WJL^	n/a
806	Hypothetical protein	2	*L.p*.^F^ *L.p*.^WJL^	n/a
894	Hypothetical protein	2	*L.p*.^F^ *L.p*.^WJL^	n/a

* *Some taxa contain more than one copy of this gene*.

*L.p.^F^, L. plantarum^F^; L.p.^WJL^, L. plantarum WJL; L.b.^F^, L. brevis^F^; L.b.^EW^, L. brevis EW*.

∧ *n/a indicates that only two taxa contain this gene*.

## Discussion

In this study, we conducted a detailed phenotypic analysis of bacteria isolated from the gut of *Drosophila* and compared these bacteria to close relatives isolated from other environments, with the objective of identifying candidate genes relevant to symbiosis in fly-associated bacteria. The fly isolates of different species varied in their response to atmospheric oxygen, capacity to utilize different carbon sources and tolerate various chemical stressors, demonstrating that bacteria with diverse phenotypic traits can colonize *Drosophila* guts. This conclusion was further supported by the capacity of bacterial isolates of non-fly origin to associate with *Drosophila*. Nevertheless, the fly and non-fly isolates differed in their impact on host traits, providing the opportunity to identify candidate genes relevant to symbiosis in fly-associated bacteria. By comparative genomic analyses, multiple genetic differences were identified, and they are predicted to include genes that contribute to the adaptation of bacteria to the gut habitat. These genes fall into three general categories: (a) metabolism, (b) putative colonization factors, and (c) stress resistance.

Metabolic interactions are at the core of many symbioses, and it is likely that this is the case with the gut microbiota of *Drosophila* as well. Several studies implicate B vitamin production by the gut microbiota as an important trait for promoting larval development in *Drosophila* (Blatch et al., 2010; Fridmann-Sirkis et al., 2014; Piper et al., 2014; Wong et al., 2014). Our results are consistent with the prediction that riboflavin is a key factor in promotion of development, as we found that *L. brevis*^F^ and *L. plantarum*^F^ can synthesize riboflavin and support faster development than conspecific isolates that cannot (Figure 1; Tables S5–S7). Additionally, a previous study found that *L. plantarum* WJL and *L. plantarum*^F^ (labeled *L. plantarum^CNW10^* therein) each promoted larval development on a low yeast diet to a greater extent than *L. plantarum*^NF^ (Storelli et al., 2011). While riboflavin biosynthesis is common to *L. brevis*^F^, *L. plantarum*^F^, and *L. plantarum* WJL, pathways for folate, nicotinamide or amino acids are variably present. Taken together, these results suggest that the ability to produce riboflavin is an important trait for the symbiotic function of Lactobacilli in *Drosophila* development. Future work should aim to verify this prediction. Riboflavin is likely a key nutrient during larval development because it serves as a precursor for FAD and FMN, cofactors for a wide range of metabolic enzymes (Barile et al., 2013).

Our analysis of the metabolic capabilities of *Acetobacter* isolates did not identify a single candidate pathway that was strongly correlated with promotion of rapid host development. However, ethanolamine utilization and fumarate reduction were two notable metabolic pathways uniquely present in *A. tropicalis*^F^, which supports the fastest larval development time of all the bacteria analyzed. Ethanolamine is an important metabolite in other gut systems, as a major breakdown product of phospholipids. It is a nutrient source exploited by successful enteric pathogens in mammals, but can also be used by non-pathogens and may be a signal by which bacteria sense the host environment (Thiennimitr et al., 2011; Kendall et al., 2012). Fumarate reduction has similarly been identified as an important metabolic process in mammalian guts, as it enables electron transport in this anaerobic environment (Fischbach and Sonnenburg, 2011). Based on BlastP searches of the NCBI nr database, it appears that genes encoding fumarate reductase are extremely rare in *Acetobacteraceae*, presumably related to the specialization of this family in highly oxidative metabolism. Gut regions of many insects are hypoxic (Karasov and Douglas, 2013). Further research to investigate the redox potential in the *Drosophila* gut is required to assess the possible significance of fumarate reduction for energy production and redox cycling by *A. tropicalis*^F^ in the *Drosophila* gut environment.

The second category of genes of interest we identified was those that may play a role in colonization of the host. Genes unique to fly isolates included a number predicted to encode cell surface proteins. A large LPXTG-motif protein unique to *L. fructivorans*^F^ bears structural similarity to biofilm adhesins in streptococci and other Gram-positive relatives (Nobbs et al., 2009; Zhou and Wu, 2009), but appears to have no homolog in the Lactobacilli (BlastP). It is interesting to note that *L. fructivorans*^F^ was less beneficial to the host than the *L. fructivorans*^NF^ strain lacking this gene. Finally, a capsular polysaccharide synthesis locus was identified as unique to *A. pomorum*^F^ (Table S3), and predicted genes in extracellular polysaccharide synthesis were identified as shared between *L. plantarum* fly isolates. Although the functional significance of these products in the *Drosophila* system remains to be established, extracellular polysaccharides produced by specific gut bacteria in mammals have been demonstrated to play critical roles in modulation of the host immune response (Round et al., 2011), and have been hypothesized to influence gut structural integrity in insects (Crotti et al., 2010).

Stress resistance was the most consistently identified function among the genes specific to fly isolates of *Acetobacter*. Specifically, two-way comparisons between fly and non-fly *Acetobacter* species identified aldehyde degradation, a superoxide dismutase and a number of efflux systems as unique to the fly strains. COGs of particular interest were identified by phylogenomic analysis, as closely related in two or more fly *Acetobacter* isolates (Table 6). Among these COGs, 7 are encoded in a single locus, including a putative oxidoreductase, aldehyde dehydrogenase, protease, and organic hydroperoxide resistance proteins (Figure 3E). Reactive oxygen species (ROS) are a major mediator of host control of the microbiota in the *Drosophila* gut as well as other animals (Ha et al., 2009; Lee and Lee, 2014; Neish and Jones, 2014). Exposure to ROS leads to the oxidation of many cellular components, with one of the most common products being protein aldehydes (Grimsrud et al., 2008). Collectively, the proteins from this locus could function to mitigate such damage. The second locus identified in *Actobacter* encodes an efflux transporter, which may also play a role in detoxification (Figure 3F).

The comparative analyses of *Acetobacer* strains did not uncover a major metabolic pathway common to fly isolates, but absent from others. Consistent with this result, the effects of all *Acetobacter* species (^F^ and ^NF^) are qualitatively similar: all *Acetobacter* species reduce development time and triglyceride when compared to *Lactobacillus*-colonized or axenic flies (Figure 1). Furthermore, nearly all of the genes identified in a recent study as required for *A. pomorum* to promote larval development (Shin et al., 2011), are components of the ethanol oxidation pathway, which is common to all acetic acid bacteria. Taken together, these studies suggest that microbiota functions may be conserved in the *Acetobacter* genus, but are potentiated by the unique genetic complement of fly isolates. Such potentiating factors could include greater resistance to stress in the host environment, as evidenced by the large number of stress resistance genes we identified as unique to fly *Acetobacter* species.

Our results raise a broader question: are fly microbiota isolates ecologically distinct from their relatives found in other environments? For adults, this distinction cannot be made based solely on the ability of the bacteria to proliferate in the host environment, as our data show non-fly species achieved abundances equivalent to or greater than fly isolates. These data contrast with those from some other symbioses, where colonization is strict determinant of ecological differentiation of symbiotic bacteria (Mandel et al., 2009; Chaston et al., 2013). An alternative mechanism of differentiation could be that fly-associated bacteria engage in a different level or type of metabolic activity in the gut compared to non-fly counterparts, facilitated by an increased resistance to stress. In support of this idea, many of the genes that differentiate between these groups are predicted to function in stress resistance, including the 2-alkenal reductase locus identified by our phylogenomic analysis, which may have been recently acquired horizontally by fly-associated *Acetobacter* species (Figure 3E). This study provides the basis for a comprehensive test of this hypothesis, which should include more bacterial isolates from wild flies and additional non-fly isolates.

In conclusion, we identified a number of compelling candidates for bacterial traits that are relevant for symbiosis in the *Drosophila* gut. These candidates form a foundation for analysis of the genetic bases of functional differences between bacteria isolated from fly and non-fly environments.

### Conflict of interest statement

The authors declare that the research was conducted in the absence of any commercial or financial relationships that could be construed as a potential conflict of interest.
