# Supplementary material for: In vivo function and comparative genomic analyses of the Drosophila gut microbiota identify candidate symbiosis factors
Source: Front Microbiol. 2014 Nov 4;5:576. doi: 10.3389/fmicb.2014.00576 (PMC4219406; doi:10.3389/fmicb.2014.00576)
Supplement: Supplementary file 2 [file Image1.PDF]

Figure S1.

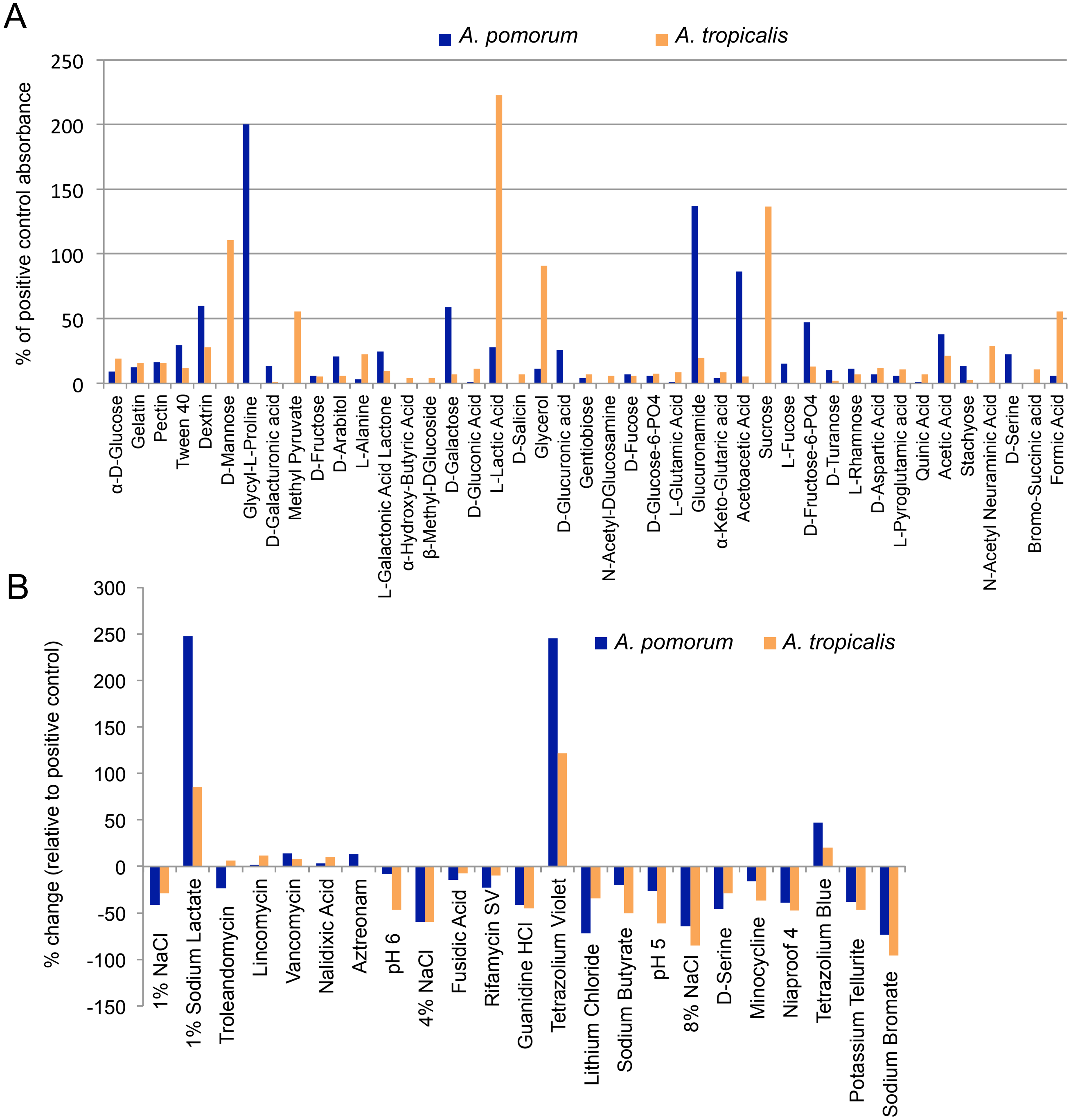

Figure S1. BioLog testing of *Acetobacter* species. *A. pomorum*^{F} and *A. tropicalis*^{F} were grown in Innoculation Fluid A (BioLog) in the Gen III MicroPlate for 36 hours, and absorbance read at 550nm. Wells producing a positive reaction, an absorbance increase of ≥15% over the negative control (well A1, which contains no carbon source), for one or more strains are reported here. (A) Absorbance values for carbon source utilization tests are shown as a percentage of the positive control (well A10, which contains an unspecified, proprietary mixture of carbon sources) after subtraction of the negative control. (B) Absorbance values for chemical resistance tests are reported as % change relative to the positive control (well A10). Thus, positive values indicate enhanced metabolic activity in the presence of the compound and negative values indicate reduced metabolic activity.

Figure S2.

A

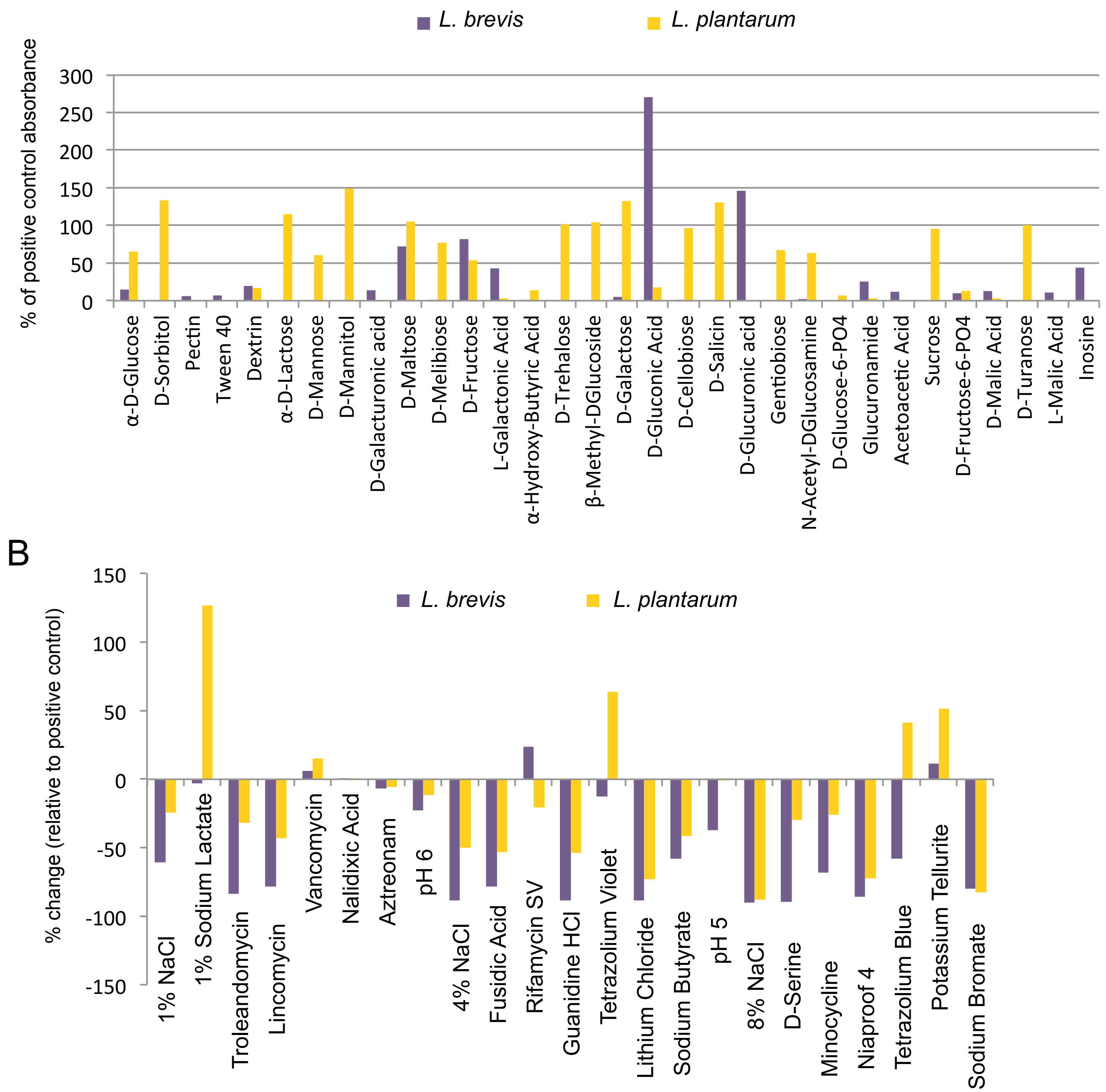

Figure S2. BioLog testing of *Lactobacillus* species. *L. brevis*^{F} and *L. plantarum*^{F} were grown in Innoculation Fluid A (BioLog) in the Gen III MicroPlate for 36 hours, and absorbance read at 550nm. Wells producing a positive reaction, an absorbance increase of ≥15% over the negative control (well A1, which contains no carbon source), for one or more strains are reported here. (A) Absorbance values for carbon source utilization tests are shown as a percentage of the positive control (well A10, which contains an unspecified, proprietary mixture of carbon sources) after subtraction of the negative control. (B) Absorbance values for chemical resistance tests are reported as % change relative to the positive control (well A10). Thus, positive values indicate enhanced metabolic activity in the presence of the compound and negative values indicate reduced metabolic activity.
